# Contribution of copy number variations to the risk of severe eating disorders

**DOI:** 10.1111/pcn.13430

**Published:** 2022-06-20

**Authors:** Itaru Kushima, Miho Imaeda, Satoshi Tanaka, Hidekazu Kato, Tomoko Oya‐Ito, Masahiro Nakatochi, Branko Aleksic, Norio Ozaki

**Affiliations:** ^1^ Department of Psychiatry Nagoya University Graduate School of Medicine Nagoya Japan; ^2^ Medical Genomics Center Nagoya University Hospital Nagoya Japan; ^3^ Department of Clinical Oncology and Chemotherapy Nagoya University Hospital Nagoya Japan; ^4^ National Hospital Organization Higashiowari National Hospital Nagoya Japan; ^5^ The Clinical Research Center, National Hospital Organization Nagoya Medical Center Nagoya Japan; ^6^ Department of Nutrition Shubun University Nagoya Japan; ^7^ Public Health Informatics Unit, Department of Integrated Health Sciences Nagoya University Graduate School of Medicine Nagoya Japan; ^8^ Institute for Glyco‐core Research (iGCORE) Nagoya University Nagoya Japan

**Keywords:** anorexia nervosa, copy number variations, eating disorders, synapses

## Abstract

**Aim:**

Eating disorders (EDs) are complex, multifactorial psychiatric conditions. Previous studies identified pathogenic copy number variations associated with NDDs (NDD‐CNVs) in ED patients. However, no statistical evidence for an association between NDD‐CNVs and EDs has been demonstrated. Therefore, we examined whether NDD‐CNVs confer risk for EDs.

**Methods:**

Using array comparative genomic hybridization (aCGH), we conducted a high‐resolution CNV analysis of 71 severe female ED patients and 1045 female controls. According to the American College of Medical Genetics guidelines, we identified NDD‐CNVs or pathogenic/likely pathogenic CNVs in NDD‐linked loci. Gene set analysis was performed to examine the involvement of synaptic dysfunction in EDs. Clinical data were retrospectively examined for ED patients with NDD‐CNVs.

**Results:**

Of the samples analyzed with aCGH, 70 severe ED patients (98.6%) and 1036 controls (99.1%) passed our quality control filtering. We obtained 189 and 2539 rare CNVs from patients and controls, respectively. NDD‐CNVs were identified in 10.0% (7/70) of patients and 2.3% (24/1036) of controls. Statistical analysis revealed a significant association between NDD‐CNVs and EDs (odds ratio = 4.69, *P* = 0.0023). NDD‐CNVs in ED patients included 45,X and deletions at *KATNAL2*, *DIP2A*, *PTPRT*, *RBFOX1*, *CNTN4*, *MACROD2*, and *FAM92B*. Four of these genes were related to synaptic function. In gene set analysis, we observed a nominally significant enrichment of rare exonic CNVs in synaptic signaling in ED patients (odds ratio = 2.55, *P* = 0.0254).

**Conclusion:**

Our study provides the first preliminary evidence that NDD‐CNVs may confer risk for severe EDs. The pathophysiology may involve synaptic dysfunction.

Eating disorders (EDs) are complex, multifactorial psychiatric conditions characterized by disordered eating behaviors, serious physical and mental health morbidity, and elevated mortality. Patients with anorexia nervosa (AN), a type of ED, show an intense fear of weight gain, abnormal body image, and weight loss behavior. Malnutrition in AN patients is strongly associated with increased risk for serious medical complications and poor prognosis. More than 70% of ED patients have psychiatric comorbidities including neurodevelopmental disorders (NDDs), mood and anxiety disorders.^1^ The etiology of EDs remains unclear, and no highly effective pharmacotherapies are available.

There is evidence not only for a substantial genetic contribution to EDs (heritability estimates up to 74% in AN)^2^ but also for shared genetic risk between EDs and other psychiatric disorders.^3^ Genome‐wide association studies based on common variants (i.e., single nucleotide polymorphisms) have revealed that AN has significant genetic correlations with schizophrenia, major depressive disorder, anxiety disorders, and obsessive–compulsive disorder.^4^, ^5^ On the other hand, rare variant studies have identified copy number variations associated with NDDs (NDD‐CNVs) in AN patients.^6^, ^7^ Most of them were large recurrent CNVs (> 500 kb): deletions at 1q21.1, 15q11.2, 15q13.3, 16p13.1 and duplications at 1q21.1, 16p11.2, 16p13.1. However, no statistical evidence for an association between NDD‐CNVs and AN has been demonstrated. One possible reason for this negative result is that they used single nucleotide polymorphism arrays that cannot reliably detect small CNVs (<100 kb). Thus, the contribution of small CNVs in EDs remains unexplored. In addition, although many risk loci for NDDs are currently known, only some of them (especially large recurrent CNVs) were investigated in previous studies. Comprehensive identification of pathogenic CNVs at NDD‐linked loci may reveal an association between pathogenic CNVs and EDs.

In this study, we conducted a high‐resolution (>10 kb) CNV analysis of severe ED patients (all females, a lifetime minimum BMI <15 kg/m^2^) and identified NDD‐CNVs at known risk loci. We focused on severe subgroup of patients because patients with severe symptoms or treatment‐resistance are more likely to carry rare deleterious variants of large effect.^8^ We found the first preliminary evidence for an association between NDD‐CNVs and severe EDs. The findings from NDD‐CNVs and gene set analysis suggest that the pathophysiology of these disorders may involve synaptic dysfunction.

## Methods

### Participants

This study was approved by the ethics committee of Nagoya University Graduate School of Medicine, and written informed consent was obtained from participants. All participants were Japanese females and recruited in the center of main island of Japan. We studied 71 severe ED patients (mean age 29.2 ± 9.4 years) and 1045 controls (mean age 37.9 ± 13.6 years). All patients required hospitalization in the psychiatric ward of Nagoya University Hospital and had a lifetime minimum BMI <15 kg/m^2^ (median: 11.3 kg/m^2^; range: 8.0–14.9 kg/m^2^). They had a clinically diagnosed history of AN restrictive type (AN‐R: n = 29), AN binge‐eating/purging type (AN‐BP: n = 36), or avoidant/restrictive food intake disorder (ARFID) (n = 6) according to the Diagnostic and Statistical Manual of Mental Disorders, 5th edition (DSM‐5). The mean age of onset was 19.5 years. Controls were selected from the general population and had no history of psychiatric disorders based upon responses to questionnaires or self‐reporting.

### CNV analysis

We performed CNV analysis using array comparative genomic hybridization (aCGH): Agilent SurePrint G3 human CGH 400k (Agilent, Santa Clara, CA). We generated CNV calls for all subjects with Nexus Copy Number software v9.0 (BioDiscovery, El Segundo, CA) using the Fast Adaptive States Segmentation Technique 2 algorithm. The following log2 ratio thresholds were set to detect CNVs in the Agilent arrays: 10–500 kb: −0.6 (deletion) and 0.4 (duplication), >500 kb: −0.4 (deletion) and 0.3 (duplication). These thresholds are much more stringent than the default thresholds of −0.2 (deletion) and 0.17 (duplication). The significance threshold to adjust the sensitivity of the segmentation algorithm was set at 1 × 10^−6^, and at least three contiguous probes were required for CNV calls. A noise‐reduction algorithm for aCGH data was used for the systematic correction of artifacts caused by GC content and fragment length.

In terms of quality control (QC), scores were calculated for each sample based on the statistical variance of the probe‐to‐probe log ratios. These QC scores showed the quality of the sample and experiment, with lower QC scores indicating higher quality results. We excluded samples with QC >0.2, gender mismatch, and excessive autosomal CNV calls (Subject QC). Next, we excluded CNV calls <10 kb, those with low probe density (<1 probe/30 kb), >70% overlap with segmental duplications, >10% overlap with CpG islands, and Call P‐value >1 × 10^−10^, and those on the Y chromosome. Then, we filtered out common CNVs (≥1% of the total sample). Finally, we obtained high‐quality rare (<1%) CNVs for all subjects. All genomic locations were given in hg18 coordinates. In our previous study, we confirmed that rare CNVs from Agilent arrays are highly accurate with a validation rate > 99%.^9^

The Wilcoxon rank‐sum test was used to compare the number and size of rare CNVs between ED patients and controls.

### Identification of NDD‐CNVs

We aimed to identify NDD‐CNVs in our sample. For this purpose, we preselected 867 loci (826 risk genes and 41 CNV loci) that are linked to NDDs (Supplementary Table 1a and b). The NDD‐linked genes were selected from the SFARI database (category 1–3) and Developmental Brain Disorder Gene Database (Tier 1–3). The association between these genes and NDDs were supported by strong genetic evidence from rare variant studies (e.g., the identification of *de novo* variants). The NDD‐linked CNV loci were selected based on our previous study.^9^ Then, we identified pathogenic or likely pathogenic CNVs in these loci according to the American College of Medical Genetics guidelines.^10^, ^11^ Briefly, pathogenic/likely pathogenic CNVs in the NDD‐linked genes included intragenic deletions and duplications overlapping with at least one exon of such genes, which would affect protein structure and function. Conversely, intronic CNVs, intragenic CNVs involving only the 3′ end of genes, and intragenic duplications overlapping with the first or last exon were not considered to be pathogenic. Intragenic duplications overlapping with the first or last exon are often not deleterious because functional gene structure may be preserved.^12^ NDD‐CNVs identified in patients were validated with quantitative real‐time PCR.

The sample size of patients was relatively small, and genetic heterogeneity of EDs was assumed to be high. Therefore, we explored an association between all NDD‐CNVs combined and EDs. The statistical significance of the association was calculated using the two‐tailed Fisher's exact test.

### Gene set analysis

To examine the involvement of synaptic dysfunction in the pathophysiology of EDs, we performed a gene set analysis using synaptic gene sets. Specifically, we evaluated whether rare exonic CNVs (both deletions and duplications) intersecting genes within a synaptic gene set are enriched in ED patients. Two synaptic gene sets were taken from SynGO^13^ and used for this analysis: synapse organization (GO:0050808, 306 genes) and synaptic signaling (GO:0099536, 193 genes). SynGO is a knowledge base that focuses on synapse‐specific ontologies, and its annotations are based on published, expert‐curated evidence.^13^ In SynGO, the selected gene sets are positioned as representative sets related to synaptic function. Two‐tailed Fisher's exact tests were used for statistical analysis. The significance level α was determined by dividing 0.05 by the number of tests for Bonferroni correction (α = 0.05 / 2 = 0.025): *P*‐values below 0.025 were considered significant, while *P*‐values between 0.025 and 0.05 were considered nominally significant.

### Phenotypic assessment

We obtained longitudinal clinical data for patients with NDD‐CNVs from medical records. The data included developmental history, age at onset, psychiatric symptoms, number of admissions, psychiatric comorbidities, premorbid IQ (JART scores), and brain imaging findings. Comorbid psychiatric disorders were assessed based on the developmental and current history and other available information obtained by interviews from patients and their families. Diagnosis was made according to the DSM‐5.

## Results

### Identification of CNVs

Of the 71 severe ED patients and 1045 controls analyzed with aCGH, 70 patients (98.6%) and 1036 controls (99.1%) passed our quality control filtering. The lifetime minimum BMI in ED patients was 11.4 kg/m^2^ (range: 8.0–14.9 kg/m^2^). We identified 2728 rare CNVs (<1%) in all subjects and their characteristics are shown in Table 1. Of these CNVs, 38% and 62% were < 50 kb and < 100 kb in size, respectively. There was no significant difference in the number and size of rare CNVs between ED patients and controls (number: *P* = 0.38, size: *P* = 0.71).

**Table 1:** Characteristics of rare CNVs

Diagnosis	ED patients	CONT	Total
Sample size (after QC)	70	1036	1106
Total number of rare CNVs	189	2539	2728
Mean number of rare CNVs per subject	2.70	2.45	2.47
Proportion of deletions	0.54	0.54	0.54
Proportion of <100 kb	0.6	0.62	0.62
Proportion of <50 kb	0.35	0.38	0.38
Median CNV size (kb)	74.1	69.6	69.6

Abbreviations: CNV, copy number variation; CONT, control; ED, eating disorder.

### Identification of NDD‐CNVs

**Fig. 1:**
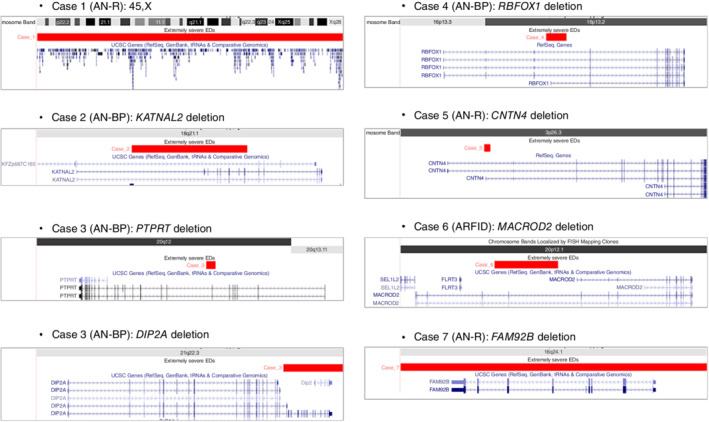
NDD‐CNVs in severe ED patients. NDD‐CNVs identified in patients with severe EDs. They include 45,X and deletions at *KATNAL2*, *PTPRT*, *DIP2A*, *RBFOX1*, *CNTN4*, *MACROD2*, and *FAM92B*. The deletions affect at least one exon of NDD genes including four synaptic genes (*PTPRT*, *DIP2A*, *RBFOX1*, and *CNTN4*). Abbreviations: AN‐BP, anorexia nervosa binge‐eating/purging type; AN‐R, anorexia nervosa restrictive type; ARFID, avoidant/restrictive food intake disorder.

Table 2 shows the identified NDD‐CNVs in the present study. We found eight NDD‐CNVs in seven ED patients (three AN‐R, three AN‐BP, and one ARFID): 45,X and deletions at *KATNAL2*, *DIP2A*, *PTPRT*, *RBFOX1*, *CNTN4*, *MACROD2*, and *FAM92B*. These deletions affected at least one exon of NDD genes (Fig. 1). The NDD‐CNVs identified in ED patients affected four synaptic genes (i.e., *PTPRT*, *DIP2A*, *RBFOX1*, and *CNTN4*). One AN‐BP patient (Case 3) had two NDD‐CNVs (deletions at *DIP2A* and *PTPRT*). In controls, we identified 24 NDD‐CNVs: 17 CNVs disrupting NDD genes and seven large recurrent CNVs. Thus, 10.0% (7/70) of ED patients and 2.3% (24/1036) of controls carried one or two NDD‐CNVs. Statistical analysis showed a significant excess of these CNVs in ED patients compared to controls (odds ratio = 4.69, *p* = .0023).

**Table 2:** List of NDD‐CNVs identified in the present study

Sample ID	Diagnosis	CNV regions (hg18)	CNV size (kb)	NDD‐CNVs
Case 1	AN‐R	chrX:239315–154882257	154643	45,X
Case 2	AN‐BP	chr18:42803418–42850905	47	*KATNAL2* del
Case 3	AN‐BP	chr20:40713672–40755445	42	*PTPRT* del
Case 3	AN‐BP	chr21:46792833–46944323	151	*DIP2A* del
Case 4	AN‐BP	chr16:6729807–6874172	144	*RBFOX1* del
Case 5	AN‐R	chr3:2249529–2272390	23	*CNTN4* del
Case 6	ARFID	chr20:14512172–14984703	473	*MACROD2* del
Case 7	AN‐R	chr16:83647246–83797767	151	*FAM92B* del
Control 1	CONT	chr16:74896665–74925155	28	*CNTNAP4* dup
Control 2	CONT	chr2:50871072–51045009	174	*NRXN1* del
Control 3	CONT	chr22:38823826–38885747	62	*TNRC6B* del
Control 4	CONT	chr15:23478243–23561947	84	*ATP10A* dup
Control 5	CONT	chrX:31663373–31824111	161	*DMD* del
Control 6	CONT	chr11:98640950–98771710	131	*CNTN5* dup
Control 7	CONT	chr15:20194004–20751393	557	15q11.2 (*NIPA1*) del
Control 8	CONT	chr16:15031188–16701937	1671	16p13.11 (*NDE1*, *MYH11*) dup
Control 9	CONT	chr22:49223470–49285901	62	*SBF1* del
Control 10	CONT	chr11:99236699–99319332	83	*CNTN5* del
Control 11	CONT	chr4:92011820–92091768	80	*CCSER1* dup
Control 12	CONT	chr15:28585517–30241239	1656	15q13.3 (*CHRNA7*, *FAN1*) dup
Control 13	CONT	chr11:99086434–105672141	6586	11q22.1‐q22.3 (*CNTN5*, *TRPC6*) del
Control 14	CONT	chr5:11422713–11441999	19	*CTNND2* del
Control 15	CONT	chr6:167194010–167252680	59	*RPS6KA2* del
Control 16	CONT	chr22:17271966–19961412	2689	22q11.21 (velocardiofacial syndrome region) dup
Control 17	CONT	chr16:21753133–22445650	693	16p12.1 (*EEF2K*, *CDR2*) del
Control 18	CONT	chr20:14905262–15154939	250	*MACROD2* del
Control 19	CONT	chr11:99319332–99414236	95	*CNTN5* dup
Control 20	CONT	chr10:56120554–56328279	208	*PCDH15* del
Control 21	CONT	chr15:20194004–20987146	793	15q11.2 (*NIPA1*) del
Control 22	CONT	chrX:6571854–7935080	1363	Xp22.31 (X‐linked ichthyosis region, *STS*) del
Control 23	CONT	chr9:119081759–119111496	30	*ASTN2* dup
Control 24	CONT	chr1:53259586–53292991	33	*SCP2* del

Abbreviations: AN‐BP, anorexia nervosa binge‐eating/purging type; AN‐R, anorexia nervosa restrictive type; ARFID, avoidant/restrictive food intake disorder; CONT, control; del, deletion; dup, duplication; NDD, neurodevelopmental disorder.

### Gene set analysis

The results of gene set analysis are shown in Table 3. We found a nominally significant enrichment of rare exonic CNVs in synaptic signaling in ED patients (odds ratio = 2.55, *P* = 0.0254).

**Table 3:** Results of gene set analysis

		% of subjects with exonic CNVs intersecting genes within gene set			
Synaptic gene sets	No of genes	ED patients	Controls	OR (95% CI)	*P*
synaptic signaling (GO:0099536)	193	11.4	4.82	2.55 (1.16, 5.60)	0.0254
synapse organization (GO:0050808)	306	11.4	6.08	1.99 (0.91, 4.34)	0.123

Abbreviations: CI, confidence interval; OR, odds ratio.

### Phenotypic assessment

Table 4 summarizes clinical data for patients with NDD‐CNVs. Age of onset of EDs was 15–23 years, and lifetime lowest BMI ranged from 10.6 to 14.6 kg/m^2^. Case 1 with 45,X had a history of language delay. Case 4 with *RBFOX1* deletion had mild intellectual disability and alcohol use disorder. Her full‐scale IQ, verbal IQ, and performance IQ were 75, 64, and 74, respectively. Although these results showed borderline intelligence, she was clinically determined to have mild intellectual disability based on the information of her developmental, educational, and life history. Two other patients (Case 5 with *CNTN4* deletion and Case 6 with *MACROD2* deletion) had a comorbidity of major depressive disorder. Three of seven patients showed cortical atrophy on brain MRI. We compared clinical variables between patients with (N = 7) and without (N = 63) NDD‐CNVs. No significant difference in age of onset, number of admissions, or premorbid IQ (*p* > .05) was found.

**Table 4:** A brief summary of clinical data for ED patients with NDD‐CNVs

Patient	NDD‐CNVs	Diagnosis	Family history of psychiatric disorders	Developmental history	Age at Onset (years)	Severe dietary restriction	Disturbed body image	Fear of gaining weight	Binge‐eating/purging	Lifetime lowest BMI (kg/m^2^)	Psychiatric comorbidity	Brain MRI
Case 1	45,X	AN‐R	−	Language delay	23	+	+	+		12.0	−	Cortical atrophy
Case 2	*KATNAL2* deletion	AN‐BP	−	Normal	20		+	+	+	12.2	−	NA
Case 3	*DIP2A* deletion *PTPRT* deletion	AN‐BP	−	Normal	22		+	+	+	11.0	−	Cortical atrophy
Case 4	*RBFOX1* deletion	AN‐BP	+	Cognitive delay	15		+	+	+	10.6	Mild ID, alcohol use disorder	Cortical atrophy
Case 5	*CNTN4* deletion	AN‐R	NA	Normal	16	+	+	+		14.6	MDD	Normal
Case 6	*MACROD2* deletion	ARFID	−	Low birth weight	20	+				11.0	MDD	NA
Case 7	*FAM92B* deletion	AN‐R	+	Normal	15	+	+	+		13.6	−	Arachnoid cyst of middle cranial fossa

Abbreviations: AN‐BP, anorexia nervosa binge‐eating/purging type; AN‐R, anorexia nervosa restrictive type; ARFID, avoidant/restrictive food intake disorder; ASD, autism spectrum disorder; BMI, body mass index; ID, intellectual disability; MDD, major depressive disorder; MRI, magnetic resonance imaging; NA, not available; NDD, neurodevelopmental disorder.

## Discussion

We provide the first evidence for an association between NDD‐CNVs and severe EDs (odds ratio = 4.69, *P* = 0.0023). This highlights an important role for CNVs in the risk for severe EDs. The NDD‐CNVs identified in patients included 45,X and deletions at *KATNAL2*, *PTPRT*, *DIP2A*, *RBFOX1*, *CNTN4*, *MACROD2*, and *FAM92B*. These genes were associated with risk of autism spectrum disorder and/or other NDDs by identification of *de novo* (loss‐of‐function) variants.^14^, ^15^, ^16^, ^17^, ^18^, ^19^ In addition, two NDD‐CNVs (*CNTN4* deletion and 45,X) were also reported in ED patients. Both deletion and duplication disrupting *CNTN4* were observed in AN patients.^6^ In a recent population‐based study, females with 45,X (Turner syndrome) were found to have twice the risk of EDs.^20^ These findings are consistent with studies showing shared genetic factors between EDs and NDDs.^3^, ^21^ Phenotypic data also showed a high rate (43%) of developmental problems in patients with NDD‐CNVs, including language delay, cognitive delay, and low birth weight. Three AN patients showed cortical atrophy on brain MRI. This was possibly caused by severe dehydration due to malnutrition.^22^

The NDD‐CNVs identified in ED patients affected four synaptic genes (*PTPRT*, *DIP2A*, *RBFOX1*, and *CNTN4*). This finding is noteworthy because few studies have linked synaptic dysfunction to the pathophysiology of EDs. *PTPRT* encodes protein tyrosine phosphatase receptor type T, is exclusively expressed in the central nervous system, and regulates synaptic formation and function.^23^, ^24^ Specifically, PTPRT regulates the expression of AMPA receptors, membrane trafficking of GluR2, GABAergic synaptic functions, and neurogenesis in the dentate gyrus. Interestingly, *Ptprt* knockout mice show reduced food intake with less body fat and are resistant to high‐fat diet‐induced obesity.^25^ DIP2A is involved in the synthesis of acetylated coenzyme A and is primarily expressed in the brain. *Dip2a*‐deficient mice exhibit abnormal spine morphogenesis, reduced synaptic transmission, and autism‐like behavior.^26^ RBFOX1 is a splicing factor that plays an important role in the regulation of the alternative splicing of large neuronal gene networks involved in brain development.^27^ RBFOX1 plays a critical role in shaping excitatory and inhibitory synaptic function and neuronal connectivity.^28^ CNTN4 has an important function related to synaptic plasticity and associative learning.^29^ *Cntn4*‐deficient mice show increased fear conditioning, which is a potential underlying mechanism of AN.^30^

In gene set analysis, we observed a nominally significant enrichment of rare exonic CNVs in synaptic signaling in ED patients. This result further suggests the possible involvement of synaptic dysfunction in severe EDs. This synaptic function was also implicated in other psychiatric disorders.^31^, ^32^

Our study has both strengths and limitations. The strengths of this study are to focus on the severe subgroup of EDs. In many complex genetic disorders, individuals with severe symptoms or treatment‐resistance are more likely to carry pathogenic variants of large effect.^8^, ^33^ Another strength is the use of high‐resolution aCGH. This allowed us to detect small CNVs (< 50 kb) including three NDD‐CNVs (deletions at *KATNAL2*, *PTPRT*, and *CNTN4*) in ED patients. The limitation is the small sample size, especially for ED patients. Therefore, our findings should be replicated in future studies with larger samples. Another limitation is that we could not confirm the inheritance pattern of NDD‐CNVs because genomic DNA from parents of ED patients was not available.

In conclusion, our study suggests that NDD‐CNVs may confer risk for severe EDs. The pathophysiology may involve synaptic dysfunction.

## Disclosure statement

M.I., S.T., H.K., T.O., M.N., and B.A. declare no conflict of interest. I.K. has received research grant from the SENSHIN Medical Research Foundation. N.O. has received research support or speakers' honoraria from, or has served as a consultant to, Sumitomo Dainippon, Eisai, Otsuka, KAITEKI, Mitsubishi Tanabe, Shionogi, Eli Lilly, Mochida, DAIICHI SANKYO, Nihon Medi‐Physics, Takeda, Meiji Seika Pharma, EA Pharma, Pfizer, MSD, Lundbeck Japan, Taisho Pharma, outside the submitted work.

## Author contributions

I.K. and N.O. designed the study. I.K., B.A., and H.K. performed the experiments. I.K. and M.N. analyzed the data. M.I., S.T., and T.O. recruited the participants and/or collected DNA samples or phenotype data. I.K. wrote the first draft of the manuscript, and the other authors commented on and refined the manuscript. All authors carefully read the manuscript and approved the final version for submission.

## Supporting information


**Supplementary Table 1a**. 826 genes linked to NDDs
**Supplementary Table 1b**. 41 CNV loci linked to NDDs

## References

[1] Treasure J , Duarte TA , Schmidt U . Eating disorders. Lancet 2020; 395: 899–911. 32171414 10.1016/S0140-6736(20)30059-3

[2] Watson HJ , Palmos AB , Hunjan A , Baker JH , Yilmaz Z , Davies HL . Genetics of eating disorders in the genome‐wide era. Psychol. Med. 2021; 51: 2287–2297. 33583449 10.1017/S0033291720005474 PMC8790815

[3] Koch SV , Larsen JT , Mouridsen SE *et al*. Autism spectrum disorder in individuals with anorexia nervosa and in their first‐ and second‐degree relatives: Danish nationwide register‐based cohort‐study. Br. J. Psychiatry 2015; 206: 401–407. 25657359 10.1192/bjp.bp.114.153221

[4] Duncan L , Yilmaz Z , Gaspar H *et al*. Significant locus and metabolic genetic correlations revealed in genome‐wide association study of anorexia nervosa. Am. J. Psychiatry 2017; 174: 850–858. 28494655 10.1176/appi.ajp.2017.16121402 PMC5581217

[5] Watson HJ , Yilmaz Z , Thornton LM *et al*. Genome‐wide association study identifies eight risk loci and implicates metabo‐psychiatric origins for anorexia nervosa. Nat. Genet. 2019; 51: 1207–1214. 31308545 10.1038/s41588-019-0439-2 PMC6779477

[6] Wang K , Zhang H , Bloss CS *et al*. A genome‐wide association study on common SNPs and rare CNVs in anorexia nervosa. Mol. Psychiatry 2011; 16: 949–959. 21079607 10.1038/mp.2010.107

[7] Yilmaz Z , Szatkiewicz JP , Crowley JJ *et al*. Exploration of large, rare copy number variants associated with psychiatric and neurodevelopmental disorders in individuals with anorexia nervosa. Psychiatr. Genet. 2017; 27: 152–158. 28368970 10.1097/YPG.0000000000000172 PMC5493193

[8] Zoghbi AW , Dhindsa RS , Goldberg TE *et al*. High‐impact rare genetic variants in severe schizophrenia. Proc. Natl. Acad. Sci. U. S. A. 2021; 118. 10.1073/pnas.2112560118 PMC8713775 34903660

[9] Kushima I , Aleksic B , Nakatochi M *et al*. Comparative analyses of copy‐number variation in autism Spectrum disorder and schizophrenia reveal etiological overlap and biological insights. Cell Rep. 2018; 24: 2838–2856. 30208311 10.1016/j.celrep.2018.08.022

[10] Riggs ER , Andersen EF , Cherry AM *et al*. Technical standards for the interpretation and reporting of constitutional copy‐number variants: A joint consensus recommendation of the American College of Medical Genetics and Genomics (ACMG) and the clinical genome resource (ClinGen). Genet. Med. 2020; 22: 245–257. 31690835 10.1038/s41436-019-0686-8 PMC7313390

[11] Brandt T , Sack LM , Arjona D *et al*. Adapting ACMG/AMP sequence variant classification guidelines for single‐gene copy number variants. Genet. Med. 2020; 22: 336–344. 31534211 10.1038/s41436-019-0655-2

[12] Truty R , Paul J , Kennemer M *et al*. Prevalence and properties of intragenic copy‐number variation in Mendelian disease genes. Genet. Med. 2019; 21: 114–123. 29895855 10.1038/s41436-018-0033-5 PMC6752305

[13] Koopmans F , van Nierop P , Andres‐Alonso M *et al*. SynGO: An evidence‐based, expert‐curated Knowledge Base for the synapse. Neuron 2019; 103: 217–34 e4. 31171447 10.1016/j.neuron.2019.05.002 PMC6764089

[14] O'Roak BJ , Vives L , Girirajan S *et al*. Sporadic autism exomes reveal a highly interconnected protein network of de novo mutations. Nature 2012; 485: 246–U136. 22495309 10.1038/nature10989 PMC3350576

[15] Yuen RK , Merico D , Cao H *et al*. Genome‐wide characteristics of de novo mutations in autism. NPJ Genom. Med. 2016; 1: 160271–1602710. 27525107 10.1038/npjgenmed.2016.27 PMC4980121

[16] Iossifov I , O'Roak BJ , Sanders SJ *et al*. The contribution of de novo coding mutations to autism spectrum disorder. Nature 2014; 515: 216–221. 25363768 10.1038/nature13908 PMC4313871

[17] Sebat J , Lakshmi B , Malhotra D *et al*. Strong association of de novo copy number mutations with autism. Science 2007; 316: 445–449. 17363630 10.1126/science.1138659 PMC2993504

[18] Schluth‐Bolard C , Diguet F , Chatron N *et al*. Whole genome paired‐end sequencing elucidates functional and phenotypic consequences of balanced chromosomal rearrangement in patients with developmental disorders. J. Med. Genet. 2019; 56: 526–535. 30923172 10.1136/jmedgenet-2018-105778

[19] Krumm N , Turner TN , Baker C *et al*. Excess of rare, inherited truncating mutations in autism. Nat. Genet. 2015; 47: 582–588. 25961944 10.1038/ng.3303 PMC4449286

[20] Bjorlin Avdic H , Butwicka A , Nordenstrom A *et al*. Neurodevelopmental and psychiatric disorders in females with Turner syndrome: A population‐based study. J Neurodev Disord. 2021; 13: 51. 34706642 10.1186/s11689-021-09399-6 PMC8554886

[21] Cross‐Disorder Group of the Psychiatric Genomics Consortium . Electronic address pmhe, cross‐disorder Group of the Psychiatric Genomics C. genomic relationships, novel loci, and pleiotropic mechanisms across eight psychiatric disorders. Cell 2019; 179: 1469–82 e11. 31835028 10.1016/j.cell.2019.11.020 PMC7077032

[22] Duning T , Kloska S , Steinstrater O , Kugel H , Heindel W , Knecht S . Dehydration confounds the assessment of brain atrophy. Neurology 2005; 64: 548–550. 15699394 10.1212/01.WNL.0000150542.16969.CC

[23] Lim SH , Kwon SK , Lee MK *et al*. Synapse formation regulated by protein tyrosine phosphatase receptor T through interaction with cell adhesion molecules and Fyn. EMBO J. 2009; 28: 3564–3578. 19816407 10.1038/emboj.2009.289 PMC2782100

[24] Lim SH , Shin S , Kim MH *et al*. Depression‐like behaviors induced by defective PTPRT activity through dysregulated synaptic functions and neurogenesis. J. Cell Sci. 2020; 133. 10.1242/jcs.243972 32938684

[25] Feng X , Scott A , Wang Y *et al*. PTPRT regulates high‐fat diet‐induced obesity and insulin resistance. PLoS One. 2014; 9: e100783. 24949727 10.1371/journal.pone.0100783 PMC4065109

[26] Ma J , Zhang LQ , He ZX *et al*. Autism candidate gene DIP2A regulates spine morphogenesis via acetylation of cortactin. PLoS Biol. 2019; 17: e3000461. 31600191 10.1371/journal.pbio.3000461 PMC6786517

[27] Li YI , Sanchez‐Pulido L , Haerty W , Ponting CP . RBFOX and PTBP1 proteins regulate the alternative splicing of micro‐exons in human brain transcripts. Genome Res. 2015; 25: 1–13. 10.1101/gr.181990.114 PMC4317164 25524026

[28] Prashad S , Gopal PP . RNA‐binding proteins in neurological development and disease. RNA Biol. 2021; 18: 972–987. 32865115 10.1080/15476286.2020.1809186 PMC8216196

[29] Oguro‐Ando A , Bamford RA , Sital W *et al*. Cntn4, a risk gene for neuropsychiatric disorders, modulates hippocampal synaptic plasticity and behavior. Transl. Psychiatry 2021; 11: 106. 33542194 10.1038/s41398-021-01223-y PMC7862349

[30] Strober M . Pathologic fear conditioning and anorexia nervosa: On the search for novel paradigms. Int. J. Eat. Disord. 2004; 35: 504–508. 15101066 10.1002/eat.20029

[31] Peng J , Zhou Y , Wang K . Multiplex gene and phenotype network to characterize shared genetic pathways of epilepsy and autism. Sci. Rep. 2021; 11: 952. 33441621 10.1038/s41598-020-78654-y PMC7806931

[32] Vevera J , Zarrei M , Hartmannova H *et al*. Rare copy number variation in extremely impulsively violent males. Genes Brain Behav. 2019; 18: e12536. 30411505 10.1111/gbb.12536

[33] Kushima I , Aleksic B , Nakatochi M *et al*. High‐resolution copy number variation analysis of schizophrenia in Japan. Mol. Psychiatry 2017; 22: 430–440. 27240532 10.1038/mp.2016.88

