# Supplementary material for: Contribution of copy number variations to the risk of severe eating disorders
Source: Psychiatry Clin Neurosci. 2022 Jun 20;76(9):423–8. doi: 10.1111/pcn.13430 (PMC9546291; doi:10.1111/pcn.13430)
Supplement: Supplementary file 1 — Supplementary Table 1a. 826 genes linked to NDDs Supplementary Table 1b. 41 CNV loci linked to NDDs [file PCN-76-423-s001.zip › PCN_13430_Supplementary Table 1a.pdf]

Supplementary Table 1a. 826 genes linked to NDDs

Genes	SFARI Gene Scoring	Developmental Brain Disorder Gene Database (Tier)
ABAT	3	-
ABCA13	3	-
ABCA7	3	-
ACE	3	-
ACHE	2	-
ACTN4	3	-
ADA	2	-
ADCY3	2	-
ADCY5	3	-
ADK	3	-
ADNP	1	1
ADORA3	3	-
ADSL	1	AR
ADSS2	3	-
AFF2	1	-
AGAP1	3	-
AGAP2	2	-
AGMO	3	-
AGO1	2	-
AGO3	3	-
AGO4	2	-
AGTR2	3	-
AHDC1	1	1
AKAP9	2	-
ALDH5A1	1	-
AMPD1	2	-
ANK2	1	1
ANK3	1	4
ANKRD11	1	1
ANKS1B	3	4
ANXA1	2	-
AP2S1	1	-
APBA2	3	-
APBB1	2	-
APH1A	2	3
ARHGAP11B	3	-
ARHGAP32	3	4
ARHGAP5	3	-
ARHGEF10	3	-
ARHGEF9	1	1
ARID1B	1	1
ARID2	3	2
ARNT2	3	-
ARX	1	2
ASAP2	2	-
ASB14	3	-
ASH1L	1	1
ASMT	3	4
ASPM	2	AR
ASTN2	2	4
ASXL1	-	1
ASXL2	-	1
ASXL3	1	1
ATP10A	2	-
ATP1A1	3	-
ATP1A3	3	-
ATP2B2	2	-
ATP6V0A2	3	-
ATRX	1	3
AUTS2	1	1
AVPR1A	2	-

Genes	SFARI Gene Scoring	Developmental Brain Disorder Gene Database (Tier)
AZGP1	3	-
BAZ2B	1	-
BCAS1	3	-
BCKDK	1	-
BCL11A	1	1
BCLAF1	-	2
BIRC6	3	3
BRAF	1	-
BRCA2	3	-
BRD4	3	-
BRINP3	3	-
BRPF1	-	1
BRSK2	1	2
BTAF1	2	-
BTRC	3	-
C15orf62	3	-
C4B	3	-
CA6	3	-
CACNA1B	3	-
CACNA1C	1	4
CACNA1D	2	-
CACNA1E	1	-
CACNA1F	3	-
CACNA1G	3	-
CACNA1H	2	-
CACNA1I	3	-
CACNA2D1	3	3
CACNA2D3	1	2
CACNB2	2	-
CADM1	3	-
CADM2	3	-
CADPS	3	-
CADPS2	3	-
CAMK2A	3	1
CAMK2B	0	1
CAPN12	3	4
CAPRIN1	1	-
CASC4	3	-
CASK	1	1
CASZ1	1	2
CBL	-	2
CC2D1A	2	AR
CCDC88C	3	-
CCDC91	3	3
CCIN	3	-
CCSER1	3	3
CCT4	2	-
CD276	3	-
CD38	3	-
CDC42BPB	2	2
CDH10	3	4
CDH11	3	-
CDH13	2	4
CDH8	3	-
CDH9	3	-
CDK13	0	1
CDKL5	1	1
CECR2	3	-
CELF4	1	-
CEP135	2	-
CEP290	3	AR
CEP41	2	-
CGNL1	2	-

Genes	SFARI Gene Scoring	Developmental Brain Disorder Gene Database (Tier)
CHAMP1	1	1
CHD1	3	-
CHD2	1	1
CHD3	1	1
CHD7	1	1
CHD8	1	1
CHM	3	-
CHMP1A	2	-
CHRM3	3	-
CHRNA7	2	3
CHRNB3	3	-
CIC	1	1
CLASP1	2	-
CLCN4	3	-
CLN8	3	-
CLTC	-	1
CMIP	3	-
CMPK2	3	-
CNGB3	3	-
CNKSR2	2	2
CNOT1	2	-
CNOT3	1	2
CNR1	2	-
CNTN3	3	-
CNTN4	2	4
CNTN5	2	-
CNTN6	2	4
CNTNAP2	2	AR
CNTNAP3	2	-
CNTNAP4	2	-
CNTNAP5	3	-
COL28A1	3	-
CPT2	3	-
CPZ	3	-
CREBBP	1	1
CSDE1	1	1
CSMD1	3	2
CSNK1E	3	-
CSNK2A1	3	-
CSNK2B	-	1
CTCF	1	1
CTNNB1	1	1
CTNND2	2	3
CTTNBP2	2	1
CUL3	1	1
CUL7	2	4
CUX1	2	-
CUX2	3	3
CX3CR1	3	-
CYFIP1	2	4
CYLC2	3	4
DAGLA	3	-
DAPP1	2	-
DCX	-	2
DDHD2	2	AR
DDX3X	1	1
DDX53	3	4
DEAF1	1	-
DENR	2	-
DHCR7	1	-
DHX57	-	2
DIP2A	1	2
DIP2C	2	-

Genes	SFARI Gene Scoring	Developmental Brain Disorder Gene Database (Tier)
DIPK2A	3	-
DISC1	2	4
DIXDC1	3	-
DLG1	3	-
DLG2	2	2
DLG4	1	1
DLGAP1	2	-
DLGAP2	3	4
DLGAP3	3	-
DLL1	3	-
DLX3	2	-
DLX6	3	-
DMD	0	3
DMPK	1	-
DMWD	3	-
DMXL2	3	AR
DNAH10	3	4
DNAH17	3	-
DNER	3	-
DNMT3A	1	1
DOCK1	3	-
DOCK4	3	-
DOCK8	2	4
DPP10	2	-
DPP3	3	3
DPP4	3	-
DPP6	3	1
DPYD	3	AR
DPYSL2	1	-
DPYSL3	3	-
DRD2	3	-
DRD3	3	-
DSCAM	1	1
DST	3	3
DUSP15	3	-
DVL3	3	-
DYDC1	3	-
DYNC1H1	1	2
DYRK1A	1	1
EBF3	1	1
EFR3A	2	-
EFTUD2	-	1
EGR3	3	-
EHMT1	1	1
EIF3G	1	-
EIF4E	3	-
EIF4G1	3	-
ELAVL2	3	2
ELAVL3	1	-
ELP4	2	-
EMSY	2	-
EN2	3	-
ENPP1	3	-
EP300	1	1
EP400	2	3
EPC2	3	-
EPHB2	3	3
EPPK1	3	-
ERBIN	2	-
ESR2	3	-
ESRRB	3	-
ETFB	2	-
EXOC5	3	-

Genes	SFARI Gene Scoring	Developmental Brain Disorder Gene Database (Tier)
EXT1	3	-
FABP5	3	-
FAM92B	2	-
FAM98C	3	-
FAN1	3	-
FAT1	3	-
FBN1	2	-
FBXO11	3	-
FBXO28	-	2
FBXO40	3	-
FCRL6	3	3
FEZF2	3	-
FGA	3	-
FHIT	3	2
FMR1	1	3
FOXG1	1	1
FOXP1	1	1
FOXP2	1	-
FRK	3	-
GABBR2	3	-
GABRA4	3	-
GABRB2	1	-
GABRB3	1	3
GALNT14	3	-
GALNT8	2	-
GATAD2B	-	1
GDA	3	-
GGNBP2	2	3
GIGYF1	1	1
GIGYF2	1	2
GLIS1	3	-
GLO1	3	-
GLRA2	3	-
GNAI1	1	-
GNAS	3	-
GNB1L	3	-
GPC3	-	1
GPC4	2	-
GPC6	3	-
GPD2	3	-
GPHN	2	4
GPR37	3	-
GPR85	3	-
GRIA1	2	-
GRIA2	1	-
GRID1	2	-
GRID2	3	4
GRID2IP	3	-
GRIK2	2	-
GRIK3	3	-
GRIK5	2	-
GRIN1	2	-
GRIN2A	2	3
GRIN2B	1	1
GRIP1	2	4
GRM5	3	-
GRM7	3	4
GTF2I	3	-
GUCY1A2	3	-
H1-4	-	1
H2BC11	3	-
HDAC4	3	3
HDAC8	0	1

Genes	SFARI Gene Scoring	Developmental Brain Disorder Gene Database (Tier)
HDLBP	1	-
HECTD4	1	2
HECW2	2	-
HIVEP2	1	1
HIVEP3	2	1
HLA-DPB1	3	-
HNRNPH2	1	-
HNRNPU	1	1
HOMER1	3	-
HRAS	1	-
HTR1B	3	-
HTR3A	3	-
HTR3C	3	-
HYDIN	3	-
ICA1	2	-
IGF1	3	-
IL1R2	3	-
IL1RAPL1	3	1
IL1RAPL2	3	-
ILF2	2	-
INPP1	3	-
INTS6	2	-
IQGAP3	3	4
IQSEC2	1	1
IRF2BPL	1	1
ITGB3	2	-
ITPR1	3	AR
JARID2	2	-
KANK1	3	4
KANSL1	1	1
KAT2B	2	-
KAT6A	2	1
KAT6B	-	1
KATNAL1	3	-
KATNAL2	1	2
KCNB1	1	-
KCNC1	3	-
KCND2	3	-
KCND3	3	-
KCNJ10	2	AR
KCNJ15	3	-
KCNK7	3	-
KCNMA1	3	-
KCNQ2	2	1
KCNQ3	1	-
KCNS3	2	2
KCTD13	3	-
KDM1B	3	-
KDM4B	3	-
KDM4C	2	-
KDM5B	1	1
KDM5C	2	1
KDM6A	2	1
KDM6B	1	1
KIAA0232	1	-
KIAA1586	2	-
KIF11	-	1
KIF13B	3	-
KIF14	2	-
KIF5C	3	-
KIRREL3	2	3
KMT2A	1	1
KMT2B	-	1

Genes	SFARI Gene Scoring	Developmental Brain Disorder Gene Database (Tier)
KMT2C	1	1
KMT2D	-	1
KMT2E	1	3
KMT5B	1	1
KRR1	3	-
KRT26	3	-
LAMA1	3	-
LAMB1	2	-
LAS1L	3	-
LDB1	1	-
LEO1	2	3
LIN7B	3	-
LMTK3	-	2
LRBA	3	4
LRFN2	3	-
LRFN5	3	-
LRP1	2	3
LRP2	3	AR
LRRC1	3	-
LRRC4	3	-
LZTR1	1	4
MACROD2	2	3
MAGEL2	1	1
MAOA	3	-
MAOB	3	-
MAP1A	1	-
MAP1B	-	2
MAPK3	3	-
MAPK8IP3	-	1
MARK1	3	-
MBD1	3	-
MBD3	3	-
MBD4	3	-
MBD5	1	1
MBOAT7	1	AR
MCM4	3	-
MCM6	3	-
MCPH1	3	3
MECP2	1	1
MED12	-	2
MED12L	3	-
MED13	1	-
MED13L	1	1
MEF2C	3	1
MEGF10	3	-
MEGF11	3	-
MEIS2	1	2
MEMO1	3	-
MET	2	-
METTL26	3	-
MFRP	2	3
MIB1	3	4
MIR137	2	-
MLANA	3	-
MOV10	-	2
MPP6	3	4
MRTFB	3	-
MSRA	-	3
MSX2	3	-
MTHFR	3	-
MTOR	2	-
MUC12	3	-
MUC4	3	-

Genes	SFARI Gene Scoring	Developmental Brain Disorder Gene Database (Tier)
MYH10	2	1
MYH4	3	-
MYLK	3	-
MYO16	3	-
MYO1E	3	-
MYO5A	2	-
MYO5C	3	-
MYO9B	2	-
MYT1L	1	1
NAA15	1	1
NAALADL2	3	4
NACC1	1	-
NAV2	2	-
NBEA	1	1
NCKAP1	1	1
NCOA1	1	-
NCOR1	2	-
NEDD9	-	2
NEXMIF	2	1
NF1	1	4
NFE2L3	2	2
NFIA	3	2
NFIB	3	2
NFIX	0	1
NINL	2	-
NIPA1	3	4
NIPA2	3	-
NIPBL	1	1
NLGN1	2	-
NLGN2	1	-
NLGN3	1	4
NLGN4X	2	3
NLGN4Y	3	-
NPAS3	-	-
NR1D1	3	-
NR2F1	3	-
NR3C2	1	2
NR4A2	1	-
NRCAM	3	-
NRP2	3	-
NRXN1	1	1
NRXN2	1	-
NRXN3	1	4
NSD1	1	1
NSD2	-	1
NTNG1	3	-
NTRK1	3	-
NTRK3	3	-
NUAK1	2	-
NUDCD2	2	-
NUDT17	-	2
NUP133	3	-
NUS1	-	1
NXPH1	3	-
OFD1	3	-
OPHN1	2	4
OR1C1	3	-
OR2T10	3	2
OR52M1	2	3
OTUD7A	2	-
OXT	3	-
OXTR	2	-
P2RX5	2	-

Genes	SFARI Gene Scoring	Developmental Brain Disorder Gene Database (Tier)
P4HA2	2	4
PACS1	1	-
PAFAH1B1	-	2
PAFAH1B2	3	-
PAH	2	AR
PAK2	2	-
PAPOLG	3	-
PARD3B	2	3
PAX5	1	-
PAX6	0	1
PBX1	3	-
PCDH10	3	-
PCDH11X	3	-
PCDH15	3	3
PCDH19	1	1
PCDH9	3	-
PCDHA1	3	-
PCDHA10	3	-
PCDHA11	3	-
PCDHA12	3	-
PCDHA13	3	-
PCDHA2	3	-
PCDHA3	3	-
PCDHA4	3	-
PCDHA5	3	-
PCDHA6	3	-
PCDHA7	3	-
PCDHA8	3	-
PCDHA9	3	-
PCDHAC1	3	-
PCM1	3	-
PDCD1	3	-
PDE1C	3	-
PDHA1	-	1
PDK2	3	-
PER1	3	-
PER2	2	-
PEX7	3	-
PHF12	1	2
PHF2	1	2
PHF21A	1	1
PHF3	1	1
PHIP	1	1
PHRF1	2	-
PITX1	3	-
PLAUR	3	-
PLCB1	2	3
PLCD4	3	-
PLN	3	-
PLXNA3	3	-
PLXNA4	2	-
PLXNB1	2	-
PNPLA7	3	-
POGZ	1	1
POLA2	3	-
POLR3A	3	3
POMGNT1	1	-
POMT1	3	-
PPFIA1	3	-
PPM1D	3	1
PPP2R1B	3	-
PPP2R5D	1	-
PPP3CA	-	1

Genes	SFARI Gene Scoring	Developmental Brain Disorder Gene Database (Tier)
PPP5C	1	-
PREX1	2	-
PRICKLE1	2	-
PRICKLE2	2	-
PRKAR1B	3	-
PRKCA	3	-
PRKCB	2	-
PRKD2	2	-
PRKDC	3	-
PRKN	2	3
PRPF39	3	-
PRR12	1	1
PRUNE2	3	-
PSD3	3	3
PSMD12	1	2
PTBP2	3	-
PTCHD1	1	1
PTCHD1-AS	2	-
PTEN	1	1
PTGS2	3	-
PTK7	1	-
PTPN11	1	-
PTPRB	3	-
PTPRC	3	-
PTPRM	-	4
PTPRT	3	-
PUF60	-	2
PURA	-	1
PXDN	3	-
PYHIN1	2	-
QRICH1	2	2
RAB11FIP5	3	-
RAB2A	2	3
RAB39B	3	3
RAB43	2	-
RAD21L1	3	-
RAI1	1	1
RALGAPB	2	1
RANBP17	2	3
RAPGEF4	3	-
RASSF5	3	-
RB1CC1	-	2
RBFOX1	2	3
RBM27	2	-
RELN	1	4
RERE	1	2
RFX3	1	-
RGS7	3	-
RIMS1	1	1
RIMS3	3	-
RNF135	3	-
RNF25	3	-
RNF38	3	-
ROBO2	2	-
RORA	0	1
RORB	1	3
RPH3A	-	2
RPL10	3	-
RPS6KA2	3	-
RPS6KA3	3	2
SAE1	2	-
SAMD11	3	-
SASH1	3	-

Genes	SFARI Gene Scoring	Developmental Brain Disorder Gene Database (Tier)
SATB1	1	-
SATB2	3	1
SBF1	2	-
SCFD2	3	-
SCN1A	1	1
SCN2A	1	1
SCN4A	3	4
SCN8A	1	3
SCN9A	2	4
SCP2	3	-
SDC2	3	-
SEMA5A	2	-
SET	2	2
SETBP1	1	1
SETD1A	3	1
SETD1B	3	3
SETD2	1	1
SETD5	1	1
SETDB1	3	-
SETDB2	3	3
SEZ6L2	3	-
SF3B1	3	-
SGSM3	3	4
SH3RF3	3	-
SHANK1	2	2
SHANK2	1	1
SHANK3	1	1
SHOX	2	4
SIN3A	1	-
SKI	1	-
SLC12A5	2	-
SLC1A1	3	-
SLC22A9	3	-
SLC24A2	3	-
SLC25A12	3	-
SLC25A39	3	-
SLC2A1	-	1
SLC35A2	-	1
SLC38A10	2	-
SLC4A10	3	3
SLC6A1	1	1
SLC6A3	2	-
SLC6A4	3	-
SLC6A8	3	2
SLC7A3	2	-
SLC7A5	2	-
SLC7A7	3	-
SLC9A6	1	1
SLC9A9	3	-
SLCO1B3	3	4
SLITRK5	2	-
SMAD4	2	-
SMARCA4	2	-
SMARCC2	1	1
SMARCE1	-	2
SMC1A	0	1
SMC3	3	-
SMG6	3	-
SMURF1	3	-
SNAP25	3	-
SND1	3	-
SNX5	2	-
SON	1	1

Genes	SFARI Gene Scoring	Developmental Brain Disorder Gene Database (Tier)
SORCS3	2	-
SOX11	-	1
SOX5	1	1
SPARCL1	2	4
SPAST	1	1
SPEN	2	2
SPP2	3	2
SRCAP	1	1
SRGAP3	3	-
SRPRA	1	-
SRRM2	-	2
SRSF11	2	2
SSPO	3	-
SSRP1	3	-
ST7	3	-
ST8SIA2	2	-
STAG1	0	1
STAG2	-	1
STX1A	3	-
STXBP1	1	1
STXBP5	2	3
STYK1	3	-
SUPT16H	2	-
SYAP1	3	-
SYN1	3	-
SYN2	3	-
SYNCRIP	3	3
SYNE1	3	4
SYNGAP1	1	1
SYP	3	-
TAF13	-	2
TAF1C	3	-
TAF6	2	-
TANC2	1	2
TAOK1	1	-
TAOK2	2	-
TBC1D31	2	-
TBC1D5	3	-
TBL1XR1	1	2
TBR1	1	1
TBX22	3	-
TCF20	1	1
TCF4	1	1
TCF7L2	1	2
TCOF1	-	1
TECTA	3	-
TERF2	2	-
TET2	2	-
THBS1	3	-
THRA	3	-
THSD7A	-	2
TLK2	1	1
TM4SF19	3	-
TM9SF4	1	-
TMEM39B	3	-
TMLHE	2	-
TNRC6B	2	1
TOP3B	3	-
TRAF7	1	-
TRAPPC2L	3	-
TRAPPC9	2	AR
TRIM23	1	-
TRIM32	3	-

Genes	SFARI Gene Scoring	Developmental Brain Disorder Gene Database (Tier)
TRIO	1	1
TRIP11	-	2
TRIP12	1	1
TRPC6	2	-
TRPM1	2	4
TRRAP	3	2
TSC1	1	2
TSC2	1	1
TSHZ3	1	-
TSPAN17	3	3
TSPAN4	3	-
TSPAN7	3	-
TSPOAP1	3	-
TTN	3	2
TUBGCP5	3	-
UBE2A	-	2
UBE3A	1	1
UBE3C	2	-
UBN2	2	2
UBR5	2	-
UIMC1	3	4
UNC13A	3	-
UNC5B	-	2
UNC5D	3	-
UNC79	2	-
UNC80	3	AR
UPF2	3	-
UPF3B	1	1
USH2A	3	-
USP15	2	-
USP45	2	-
USP7	2	1
USP9X	0	1
USP9Y	3	-
VEZF1	1	-
VIL1	2	4
VIPR2	-	-
VPS13B	1	AR
VSIG4	3	-
WAC	1	1
WASF1	0	1
WDFY3	1	1
WDFY4	2	-
WDR45	-	1
WNK3	3	-
WWOX	2	AR
WWP1	3	-
XPO1	3	-
XRCC6	3	-
YEATS2	3	-
YTHDC1	-	2
YTHDC2	3	-
YWHAE	3	-
YWHAG	3	-
ZBTB18	-	1
ZBTB20	1	-
ZC3H11A	3	-
ZC3H4	2	-
ZEB2	-	1
ZFYVE26	3	3
ZMIZ1	2	-
ZMYM6	-	2
ZMYND11	2	1

Genes	SFARI Gene Scoring	Developmental Brain Disorder Gene Database (Tier)
ZNF292	1	1
ZNF462	1	1
ZNF517	3	-
ZNF548	3	-
ZNF626	3	-
ZNF713	3	-
ZNF774	3	4
ZNF804A	2	4
ZWILCH	3	2
