# Supplementary material for: Contribution of copy number variations to the risk of severe eating disorders
Source: Psychiatry Clin Neurosci. 2022 Jun 20;76(9):423–8. doi: 10.1111/pcn.13430 (PMC9546291; doi:10.1111/pcn.13430)
Supplement: Supplementary file 1 — Supplementary Table 1a. 826 genes linked to NDDs Supplementary Table 1b. 41 CNV loci linked to NDDs [file PCN-76-423-s001.zip › PCN_13430_Supplementary Table 1b.pdf]

Supplementary Table 1b. 41 CNV loci linked to NDDs

CNV	CNV type	Genomic coordinate (hg18)
1p36 (GABRD)	Deletion	chr1:1-27799098
1q21.1 (thrombocytopenia-absent radius syndrome region, HFE2)	Deletion/Duplication	chr1:144100355-144544421
1q21.1 (GJA5)	Deletion/Duplication	chr1:145044206-145860738
2q11.2 (LMAN2L, ARID5A)	Deletion	chr2:96090475-97039464
2q12.2-q12.3 (ST6GAL2)	Deletion	chr2:106462888-107807888
2q21.1 (ARHGEF4)	Deletion/Duplication	chr2:131205043-131633043
2q37 (HDAC4)	Deletion	chr2:239370398-242119088
3q29 (DLG1, PAK2)	Deletion/Duplication	chr3:197230268-198839268
4p16.3 (Wolf-Hirschhorn syndrome region, WHSC1)	Deletion	chr4:1-4552628
5q35.2-q35.3 (Sotos syndrome region, NSD1)	Deletion	chr5:175650609-176989607
7q11.23 (Williams-Beuren syndrome region, ELN, GTF2I)	Deletion/Duplication	chr7:72380939-73779274
8p23.1 (SOX7, CLDN23)	Deletion/Duplication	chr8:8130932-11929918
9q34.3 (EHMT1)	Deletion	chr9:136950667-140199273
10q11.21-q11.23 (CHAT, SLC18A3)	Deletion/Duplication	chr10:48640964-51409766
10q23.1-q23.2 (NRG3, GRID1)	Deletion	chr10:81950736-88789737
11p11.2 (Potocki-Shaffer syndrome region, EXT2, ALX4)	Deletion	chr11:43940126-46019127
12q14.3-q15 (GRIP1, HMGA2)	Deletion	chr12:63360047-66929047
13q12.12 (SACS)	Deletion	chr13:22440139-23789138
15q11.2 (NIPA1)	Deletion	chr15:20350432-20639509
15q11.2-q13.1 (Prader-Willi/Angelman syndrome region, UBE3A)	Deletion/Duplication	chr15:21145588-26726187
15q13.1-q13.2 (APBA2)	Deletion/Duplication	chr15:26806187-28152495
15q13.3 (CHRNA7, FAN1)	Deletion/Duplication	chr15:28700495-30269493
15q24.1-q24.2 (SEMA7A, ARID3B)	Deletion/Duplication	chr15:72171394-73356711
15q25.2 (HOMER2, BNC1)	Deletion	chr15:80980033-82529756
15q26.3 (IGF1R)	Duplication	chr15:97180752-100338726
16p13.11 (NDE1, MYH11)	Deletion/Duplication	chr16:15320358-16293358
16p12.2-p11.2 (PRKCB)	Deletion/Duplication	chr16:21260822-29349822
16p12.1 (EEF2K, CDR2)	Deletion/Duplication	chr16:21850822-22369822
16p11.2 distal (SH2B1, ATP2A1)	Deletion	chr16:28680822-29019822
16p11.2 (KCTD13, TBX6)	Deletion/Duplication	chr16:29560822-30109822
17p13.3 (YWHAE, PAFAH1B1)	Deletion	chr17:50791-2539044
17p11.2 (Smith-Magenis syndrome/Potocki-Lupski syndrome region, RAI1)	Deletion/Duplication	chr17:16650001-20420000
17q11.2 (NF1)	Deletion	chr17:26190144-27239132
17q12 (renal cysts and diabetes syndrome region, HNF1B)	Deletion	chr17:31891927-33322735
17q21.31 (MAPT)	Deletion/Duplication	chr17:41060149-41539149
22q11.21 (velocardiofacial syndrome region, COMT, TBX1)	Deletion/Duplication	chr22:17275513-18691523
22q11.21 (CRKL, SNAP29)	Deletion/Duplication	chr22:19031291-19824289
22q11.22-q11.23 (BCR, RAB36)	Deletion	chr22:21310643-21992523
22q11.23 (ADORA2A)	Deletion/Duplication	chr22:21990187-23369967
Xp22.31 (X-linked ichthyosis region, STS)	Deletion	chrX:6400041-8099041
whole chrX	Deletion/Duplication	chrX:1-154913754
